# Critical Decline of the Eastern Caribbean Sperm Whale Population

**DOI:** 10.1371/journal.pone.0162019

**Published:** 2016-10-05

**Authors:** Shane Gero, Hal Whitehead

**Affiliations:** 1 Zoophysiology, Institute for Bioscience, Aarhus University, Aarhus, Denmark; 2 Dept. of Biology, Dalhousie University, Halifax, Canada; Institute of Deep-sea Science and Engineering, Chinese Academy of Sciences, CHINA

## Abstract

Sperm whale (*Physeter macrocephalus*) populations were expected to rebuild following the end of commercial whaling. We document the decline of the population in the eastern Caribbean by tracing demographic changes of well-studied social units. We address hypotheses that, over a ten-year period of dedicated effort (2005–2015), unit size, numbers of calves and/or calving rates have each declined. Across 16 units, the number of adults decreased in 12 units, increased in two, and showed no change in two. The number of adults per unit decreased at -0.195 individuals/yr (95% CI: -0.080 to -0.310; P = 0.001). The number of calves also declined, but the decline was not significant. This negative trend of -4.5% per year in unit size started in about 2010, with numbers being fairly stable until then. There are several natural and anthropogenic threats, but no well-substantiated cause for the decline.

## Introduction

When a well-defined existential threat is removed, we expect population recovery. Following the 1986 moratorium on commercial whaling, some populations of heavily-exploited species increased, but some did not [1]. Sperm whales (*Physeter macrocephalus*) were expected to increase [2], but there is little clear evidence for regrowth [3,4]. There are a range of current threats that may be affecting population growth rates [4]. Sperm whales have an especially low rate of increase [5], giving threats with quite small proximate impacts (on, say, feeding, reproduction or mortality) population-level salience, and also making the detection of population trends challenging. Thus their status is generally uncertain as very small rates of change (~±1–3% per year), which are extremely hard to identify using most cetacean population assessment methods, can be of major significance.

The world’s best-studied sperm whale population is that which uses waters near the volcanic islands of the Lesser Antilles in the eastern Caribbean [6]. Their social [6–9] and vocal [10–14] behaviour has been characterised in detail and their population monitored at the individual level for over a decade [15–18]. Female and immature sperm whale live in social units that are matrilineally based and have largely stable membership, other than birth, death and dispersal of adolescent males [19,20]. Off the island of Dominica in the eastern Caribbean, members of 17 social units have been repeatedly photoidentified since 2005 [20], with many units being documented as far back as 1984 from opportunistic data collected off seven islands in the Lesser Antilles [across a linear distance of ~670 km, 17]. Although there are occasional encounters with less-known units [20], the presence of sperm whales off Dominica is dominated by units that are well known by the standards of cetacean demographic studies.

This study was prompted first by observations in 2014 and 2015 that some of the well-known units were reduced in size; and secondly, by the conflicting results of our recent population study [9]. Mark-recapture analysis indicated a population increasing at +3.4% per year, and contrasted strongly with a parallel life history stage-based estimate of the projected rate of increase for the same population of −2.7% per year. We suggested that immigration from surrounding regions coupled with a high local mortality could explain this discrepancy, making the study area an effective attractive sink [15].

Here, we address the hypotheses that, over the ten-year period of dedicated effort (2005–2015), the well-studied units have declined in membership, and that numbers of calves and/or calving rates have declined.

## Methods

Photo-identifications of sperm whale adults [21] and calves [20] were taken off the islands of the Lesser Antilles, particularly Dominica and Guadeloupe, between 2005 and 2012 as described by Gero et al. [20], with the addition of data from 2014 and 2015 which were collected using similar methods (S1 and S2 Tables). Mature males were excluded from analyses because they are not permanent members of any unit, and, being primarily based in high-latitude waters, are not part of the eastern Caribbean population itself.

We allocated individuals to units as in [20], and then considered units only in those years in which there were a total of at least 30 photoidentifications of members of the unit by the primary research group, and there was no ambiguity about whether individuals were adults or calves. The resultant data set (numbers of adults and calves in each unit each year) is as in Table 1. The probability that an individual in a unit with 7 members (mean unit size [20]) is identified in at least one of 30 identifications of the unit in a year is 0.99 [1-(1-1/7)^30^], so that our estimates of unit size in the different years are extremely precise. Specifically, encounters off Dominica are almost always of a single unit of whales, such that there are only 7 individual whales in the surrounding kilometers. As such, during encounters each individual whale is generally identified at a minimum of every other surfacing (every two hours), if not every surfacing (each hour) [see 20]. As a result, for these well-studied units, membership is easily established for each year in which there was sufficient time spent with the unit. It is, therefore, very unlikely that members of units are missed in years with 30 or more identifications of the unit.

**Table 1:** Number of adults (plus calves) identified in each year for each social unit, where data were sufficient.

Unit	2005	2006	2007	2008	2009	2010	2011	2012	2014	2015
**A**	7(+2C)			8(+2C)	7(+1C)	7(+4C)			3(+0C)	6(+1C)
**C**	6(+0C)	6(+1C)								
**D**				5(+2C)	5(+2C)	3(+2C)	3(+2C)		2(+2C)	
**F**	6(+1C)	5(+1C)	5(+1C)	5(+2C)	5(+2C)	5(+2C)	3(+2C)	2(+2C)		2(+2C)
**G**				3(+2C)		3(+1C)				
**I**				3(+1C)	2(+1C)					
**J**			4(+0C)	4(+0C)	4(+0C)	4(+1C)	4(+1C)		3(+0C)	3(+0C)
**K**				4(+2C)	2(+0C)			4(+0C)	1(+0C)	
**N**					4(+1C)	6(+2C)		6(+2C)		3(+0C)
**P**				8(+3C)	5(+0C)		8(+0C)	5(+0C)		
**Q**		1(+1C)		7(+2C)	4(+2C)		4(+0C)	1(+0C)		
**R**	6(+1C)			6(+1C)	3(+0C)		6(+0C)	5(+0C)	4(+1C)	5(+3C)
**S**	3(+2C)	2(+1C)			3(+0C)		3(+0C)	3(+0C)	3(+0C)	3(+0C)
**T**				7(+1C)	2(+0C)	6(+2C)	6(+2C)			
**U**				3(+1C)	3(+1C)	4(+0C)	4(+0C)	2(+0C)		3(+0C)
**V**						9(+3C)	5(+1C)			

For each unit in each year, we calculated four measures: number of adults, number of calves, total size (adults plus calves), and calf rate (the number of calves in the population relative to the number of adults, *i*.*e*. calves/adults). For each measure and each unit, we regressed the measure against year to give a trend estimate. The overall trend was estimated from a mixed effects model with the measure for a unit in a particular year being predicted by the continuous independent variable year for the slope and the random grouping variable unit for the intercept. The significance of the trend was measured in two ways, representing two rather different null hypotheses:

- there was no overall trend in the measure over time, using the significance of the trend parameter in the mixed effects model;
- units were as likely to have increased as decreased in the measure over time, using a sign test on the trends for the different units.

**Fig 1:**
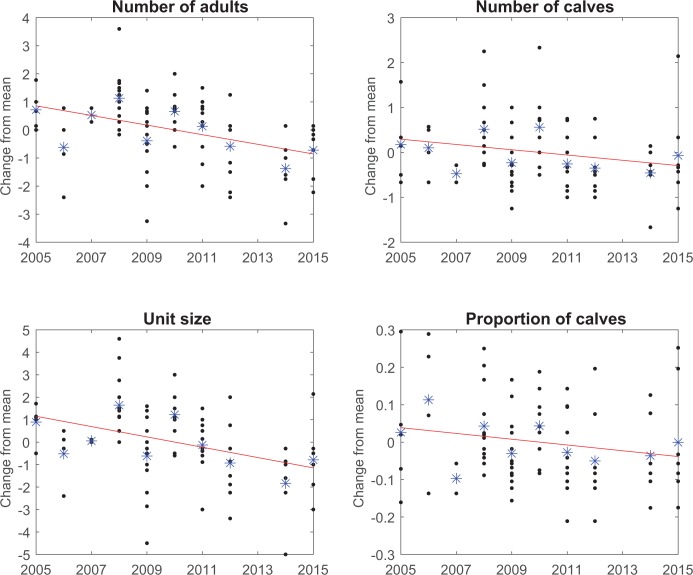
For number of adults, calves, total unit size and proportion of calves, the residual from the unit mean (over years) is plotted against the study year (dots). Also shown are the means for each year over units (star) and a regression through the residuals (line).

Although the observations that generated this quantitative study were an apparent decline of unit size, we used two-sided tests as the hypothesis was developed during the same field observations that generated the photoidentification data, and two-sided tests are more conservative. As sperm whale reproduction has a broad seasonal component [22], we checked whether the mean (over units) of the difference between the number of calves, or the calf rate, observed in any year and the overall mean for that unit (the “*”‘s in Fig 1) was related to the Julian date of the midpoint of the field season (S1 Table). There was no significant correlation of either mean residual number of calves, or mean residual calf rate, with the midpoint of the field season (P = 0.5502, P = 0.2713 respectively).

We estimated survival within these well-studied units using maximum likelihood (S5 Table). We also quantify the sighting trend of rarely-seen individuals and updated the overall mark-recapture analysis described in [15] to include 2014 and 2015 data (S4 and S5 Tables; S1 Fig).

### Ethical Statement

Data collected in Dominica under scientific research permits: SCR 013/05-02, RP-2/12 IW-1, RP-09/014 IW-1, RP-01/079W-2, RP-03/059W-4, P-122/4W-2, and P-40/2W-7. Field protocols were approved by the University Committee on Laboratory Animals of Dalhousie University.

## Results

There were 16 units with two or more reliable estimates of size in different years between 2005 and 2015 (range: 2–9 years). Of those, the number of adults decreased in 12 units, increased in 2 units, and showed no change in two (Table 2). Overall, the number of adults decreased at a rate of -0.195 individuals/yr (95% CI: -0.080 to -0.310). This trend and the sign test were both significant (P = 0.0010, P = 0.0012 respectively). The number of calves also declined, but at a lower and less significant rate, while the total unit size showed a strong and significant decline (Table 2; Fig 1). The data also showed a decline in the proportion of calves over the study period, but this was not statistically significant (Table 2). These results were almost identical if only the 12 units identified with four or more reliable estimates of size in different years were included (S3 Table). If divided by the mean number of adults, calves or both we get percentage decreases of about -4.5% per year in the numbers of adults, calves and total population size. It is apparent in Fig 1 that these negative trends start in about 2010, with numbers being fairly stable until then. The overall mark-recapture analysis (S4 Table) generally supports this trend; however this method has less power to detect trends than our principal analysis (Table 2; Fig 1). This is because trends are much more apparent in the known membership of well-studied units than in the population as a whole, which includes the confounding influence of members of rarely-sighted units.

**Table 2:** Trends for each unit (from linear regression) and overall (from linear mixed effects model), together with the significance tests of overall trend and the sign test. Means over all years, overall percent trends, and 95% confidence intervals.

	Trend per year in			
Unit	Adults	Calves	Adults+Calves	Calves/Adults
A	-0.315	-0.165	-0.480	-0.015
C	0.000	1.000	1.000	0.143
D	-0.528	0.000	-0.528	0.037
F	-0.420	0.122	-0.299	0.041
G	0.000	-0.500	-0.500	-0.075
I	-1.000	0.000	-1.000	0.083
J	-0.146	-0.003	-0.149	-0.001
K	-0.275	-0.242	-0.516	-0.040
N	-0.262	-0.214	-0.476	-0.036
P	-0.300	-0.600	-0.900	-0.055
Q	-0.105	-0.263	-0.368	-0.085
R	-0.109	0.116	0.008	0.016
S	0.049	-0.170	-0.121	-0.041
T	0.100	0.500	0.600	0.062
U	-0.059	-0.151	-0.211	-0.038
V	-4.000	-2.000	-6.000	-0.083
All:	-0.195	-0.063	-0.258	-0.009
95% CI	(-0.310–0.080)	(-0.136 0.010)	(-0.415–0.100)	(-0.021 0.002)
p (Trend)	0.001	0.088	0.002	0.119
p (Sign test)	0.001	0.074	0.001	0.134
Mean	4.38	1.06	5.44	0.176
Trend (%)	-4.45	-5.95	-4.74	-5.21
95% CI	(-7.09–1.82)	(-12.82 0.92)	(-7.64–1.85)	(-11.79 1.37)

**Fig 2:**
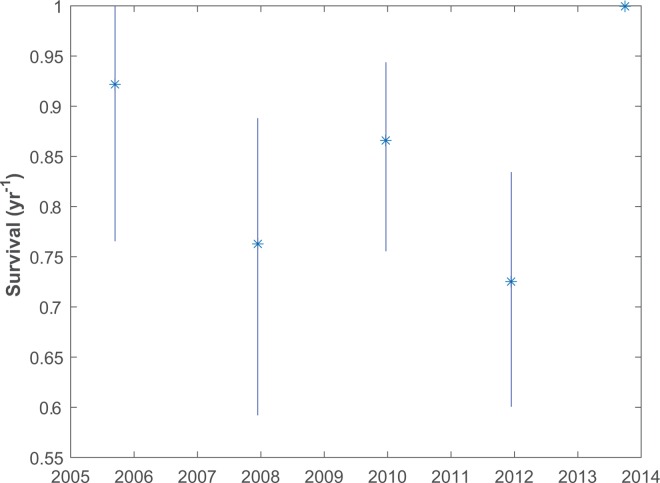
Maximum-likelihood estimates of survival rates for well-studied units off Dominica. Confidence intervals are from 1000 bootstrap replicate samples of units.

Biannual maximum-likelihood estimates of survival (including dispersal of maturing males, and infrequent changes of unit membership) indicate high mortality in the middle years of the study, roughly 2008–2013 (Fig 2). The perfect survival for 2014–2015 is based upon the 13 individuals identified in both 2014 and 2015 from 4 units, and no adults from these units being sighted in 2014 but not 2015.

## Discussion

Our analyses indicate a population under threat, perhaps in crisis. The numbers of animals in well-studied units has shown a startling decline, seemingly caused by a substantial drop in survival starting in about 2008. Reproductive rates also seem to be falling. If overall trends continue, by 2030 there will be very few animals left. Our earlier analyses [15] suggested that an influx of new whales was compensating for the demographic decline of the residents. But, off Dominica, the proportion of rarely-seen animals has also declined since about 2012 (S1 Fig), indicating that this source of population rescue is drying up. It is unlikely that what we have documented is a reduction in abundance of whales in the study area off Dominica relative to those in the habitat of the population as a whole, e.g., [23]. Given general long-term stability of units [19,20], this could only have occurred if the sperm whales’ social structure broke down causing fission of virtually all of the units. Fission of units has only been observed once in over 30 years of research in the Pacific and never in the 11 years off Dominica [19,20]; therefore it seems unlikely that fission of the vast majority of units studied here over such a short period of time is the mechanism driving this trend.

An analysis of defecation rates—used as a proxy for feeding success in sperm whales—found no decline over the study period, and a generally higher rate than studies in other regions (S2 Fig). This strongly suggests that lack of food, which could be caused by environmental changes or a density-dependent response following population rebound, is not implicated. However, the Eastern Caribbean population is subject to a number of anthropogenic threats that have proven to be fatal for this or other cetacean species, including:

1) Ship Strikes: Collisions with ships can directly impact the viability of a species (e.g. North Atlantic right whales, *Eubalaena glacialis* [24]) or subpopulations of a species (e.g. sperm whales in the Mediterranean Sea [25]). This risk is particularly acute in the Caribbean where the vast majority of goods are imported and there are high levels of marine traffic surrounding islands. Additionally, inter-island transits by high speed (>25 knots) ferries are increasing. These have proven to be a significant source of mortality for sperm whales off the Canary Islands [26,27], a similar deep-water archipelago. The eastern Caribbean is among the most urban parts of sperm whale habitat, routes of high-speed ferries, cruise ships, and other shipping pass directly through the areas used by sperm whales off the islands.
2) Tourism: Traditional vessel-based whale watching has been a growth industry in the Caribbean since the early 1990’s and Dominica was an early leader [28]. In addition, since at least 2008, several commercial swim-with-whales operations focused on sperm whales have rapidly expanded increasing both the pressure on the individual whales and the amount of time whales encounter vessels. Both of these industries remain poorly regulated in much of the eastern Caribbean.
3) Fishing: While the majority of fishing effort in the eastern Caribbean uses small-scale light gear, such as Fish Attraction Devices (FAD) and gillnets, entanglements have previously caused sperm whale fatalities [29]. There were two reported entanglements in 2015 involving sperm whales (C. Rinaldi, pers. comm. and S. Gero, unpublished). Detection rates for entangled cetaceans are low, perhaps 8–10% [30], and may well be less in the eastern Caribbean where search effort is low. Thus 20 or more sperm whale entanglements (not necessarily all fatal) may have occurred in the eastern Caribbean in 2015.
4) Research: The research required to document this decline by necessity increased the whales’ overall exposure to small vessels. However, our methods have predominantly been non-invasive, been consistent since the start of the project in 2005, and research vessels have been shown to cause less disturbance to the focal animals than whale watching vessels [31,32].
5) Pollutants: Both physical and chemical pollutants create increased mortality for cetaceans. Marine debris has been cited as among the principal threats to marine mammals in the Caribbean and ingestion can prove fatal for sperm whales [33]. Organochlorines commonly used in agriculture, such as chlordecon used in banana plantations, have been documented in coastal marine fish and lobster species in the Caribbean [34]. Analysis of sperm whale tissue biopsies for pollutants and heavy metals using existing sperm whale biopsy samples is currently underway.
6) Ocean Noise: Whales rely on their acoustic modality to communicate, navigate, and forage. Changes in their acoustic environment may have both chronic and acute impacts, including increased stress [35], masking of communication and echolocation [36,37], and other behavioural responses [38–40].

There are also potential natural causes of the decline including:

7) Disease: Successful carcass recovery of pelagic cetaceans is very low (around 3% for sperm whales generally [41]). Without detailed analysis of carcasses or biopsy samples it is difficult to determine the impact of natural illnesses.
8) Predation: While the threat of predation from orcas (*Orcinus orca*) is thought to be higher in the Pacific [42], recent observations suggest predation occurs in the Gulf of Mexico [43], around the Caribbean [44], and off Dominica (S.Gero, unpublished data).

Currently, none of these anthropogenic or natural causes can be ruled out; and the documented decline may be the result of a complex combination of threats. Unless the effects we document are the results of a temporary factor operating primarily during 2009–2014, or a statistically-unlikely burst of “natural” mortality during the same period, we expect the decline to continue in the absence of effective mitigation of ongoing threats.

## Conclusions

Detailed individual-identification data document a decline in the eastern Caribbean sperm whales. These animals form a small (<300 adults; S4 Table) population that is largely isolated (no photoidentification matches with well-studied populations in the Gulf of Mexico, the Sargasso Sea, and the Bahamas [15,17]) and behaviourally distinctive, at least in vocal repertoire and rates of allosuckling [7,11]. If extirpated, this population cannot be replaced. The data presented here call for a regional assessment of this population by the International Union for Conservation of Nature, urgent assessment of likely causes for its decline, and mitigation measures to be put in place.

## Supporting Information

S1 Fig Sighting trend of rarely-seen animals. Number of individuals identified from often-seen social units (units identified with a good sighting record in four or more years during 2005–2015), from occasionally-seen units (units identified with a good sighting record in two years during 2005–2015; there were no units identified in just three years), and rarely-seen animals (social unit identified in less than two years 2005–2015, or not assigned to units) in each year from 2005–2015. The number of days at sea by the dedicated research group in each year is given above each bar.
(DOCX)

S2 Fig Defecation rates. Defecation rates (number of defecations at fluke-ups divided by number of fluke-ups observed) for years with more than 50 observations. Bars represent approximate 95% confidence intervals from binomial distribution.
(DOCX)

S1 Table Field Effort. Field effort across years.
(DOCX)

S2 Table Supplementary Data. Details of field projects contributing opportunistic data.
(DOCX)

S3 Table Alternative methods and impacts on results. Trends in numbers of adults, calves, adults+calves and calves/adults for each unit for which we had reliable estimates in 4 or more years (from linear regression) and overall (from linear mixed effects model), together with results of two-sided tests of null hypotheses that the overall trend is zero, and that units were equally likely to increase as decrease in size during the study period (sign test). The final three lines give the mean value of each measure over all years and units with available data, the overall percent trend and 95% confidence intervals.
(DOCX)

S4 Table Overall mark-recapture population trend. Mark-recapture models fit to identifications of adult sperm whales in the eastern Caribbean, 1984–2015 sorted by decreasing ΔAIC.
(DOCX)

S5 Table Survival of well-studied units using maximum likelihood. Models of Survival in best-studied social units, 2005–2015.
(DOCX)
