# Supplementary material for: Critical Decline of the Eastern Caribbean Sperm Whale Population
Source: PLoS One. 2016 Oct 5;11(10):e0162019. doi: 10.1371/journal.pone.0162019 (PMC5051958; doi:10.1371/journal.pone.0162019)
Supplement: S1 Fig — Number of individuals identified from often-seen social units (units identified with a good sighting record in four or more years during 2005–2015), from occasionally-seen units (units identified with a good sighting record in two years during 2005–2015; there were no units identified in just three years), and rarely-seen animals (social unit identified in less than two years 2005–2015, or not assigned to units) in each year from 2005–2015. The number of days at sea by the dedicated research group in each year is given above each bar. (DOCX) [file pone.0162019.s001.docx]

## **Sighting trend of rarely-seen animals**

In S1 Fig., we plot, for each study year, the number of individuals identified (by any research group) from often-seen social units (units identified with a good sighting record in four or more years during 2005-2015), from occasionally-seen social units (units identified with a good sighting record in two years during 2005-2015; there were no units identified in just three years), and rarely-seen animals (social unit identified in less than two years 2005-2015, or not assigned to units). Mature and maturing males were excluded. The proportion of animals from occasionally-seen or rarely-seen units appears to decline during the study period. This is borne out by a negative (-0.134) and significant (P=0.034; after correction for overdispersion) logistic regression coefficient. The proportion of animals just from rarely-seen units also had a negative logistic regression coefficient (-0.083) but was not significantly different from zero (P=0.273; after correction for overdispersion).


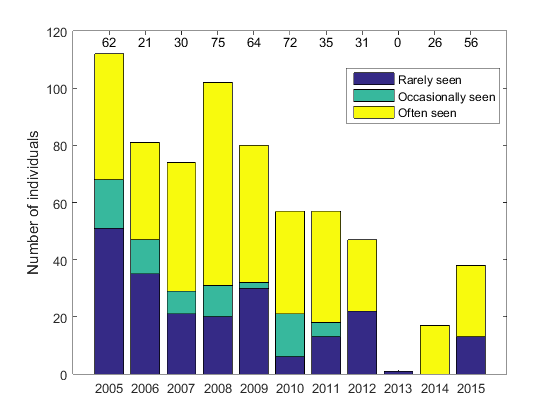


S1 Fig. - Number of individuals identified from often-seen social units (units identified with a good sighting record in four or more years during 2005-2015), from occasionally-seen units (units identified with a good sighting record in two years during 2005-2015; there were no units identified in just three years), and rarely-seen animals (social unit identified in less than two years 2005-2015, or not assigned to units) in each year from 2005-2015. The number of days at sea by the dedicated research group in each year is given above each bar.
