# Supplementary material for: Critical Decline of the Eastern Caribbean Sperm Whale Population
Source: PLoS One. 2016 Oct 5;11(10):e0162019. doi: 10.1371/journal.pone.0162019 (PMC5051958; doi:10.1371/journal.pone.0162019)
Supplement: S2 Fig — Defecation rates (number of defecations at fluke-ups divided by number of fluke-ups observed) for years with more than 50 observations. Bars represent approximate 95% confidence intervals from binomial distribution. (DOCX) [file pone.0162019.s002.docx]

**Defecation Rates:**

To investigate the possibility that feeding success may have changed, we calculated defecation rates - number of observed defecations at fluke-ups as a proportion of fluke-ups which were observed for possible defecations [1] - for those years with greater than 50 observed fluke-ups. Defecation rates are used as a proxy for feeding success in sperm whales [1].


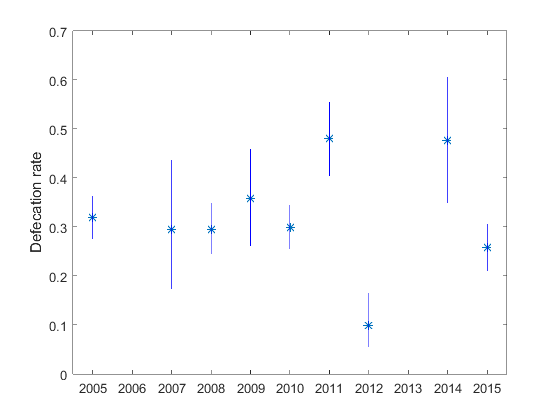
Defecation rates off Dominica, do not systematically decline over the study period and are generally high with a median of about 0.3 defecations/fluke-up (S2 Fig). Defecation rates measured here are roughly twice the median for studies of female sperm whales in the Pacific [2] or the neighbouring Sargasso Sea in the western North Atlantic [3]. Thus it seems unlikely that poor feeding success has been an important issue for the eastern Caribbean sperm whales over the study period.

S2 Fig. - Defecation rates (number of defecations at fluke-ups divided by number of fluke-ups observed) for years with more than 50 observations. Bars represent approximate 95% confidence intervals from binomial distribution.

**References:**

1. Whitehead H. Variation in the feeding success of sperm whales: Temporal scale, spatial scale and relationship to migrations. J Anim Ecol. 1996;65: 429–438.

2. Whitehead H, Rendell L. Movements, habitat use and feeding success of cultural clans of South Pacific sperm whales. J Anim Ecol. 2004;73: 190–196.

3. Gero S, Engelhaupt D, Rendell L, Whitehead H. Who Cares? Between-group variation in alloparental caregiving in sperm whales. Behav Ecol. 2009;20: 838–843.
