# Supplementary material for: Critical Decline of the Eastern Caribbean Sperm Whale Population
Source: PLoS One. 2016 Oct 5;11(10):e0162019. doi: 10.1371/journal.pone.0162019 (PMC5051958; doi:10.1371/journal.pone.0162019)
Supplement: S1 Table — Field effort across years. (DOCX) [file pone.0162019.s003.docx]

**Field Effort:**

Field work undertaken during directed research through Dalhousie University (2005-2012) and Aarhus University (2014-2015) totaled 3660 hours with whales on 402 days across 472 days of effort from 2005-2015 on one of four platforms: a dedicated 12m auxiliary sailing vessel, a dedicated 5m outboard skiff, a dedicated 11m outboard rigid-hull inflatable (RHIB) or an 18m whale-watch vessel. Effort is broken down by year and platform in Table S1.

Table S1: Field Effort across years

| **Year** | **Start Date** | **End Date** | **Days Effort** | **Platform** |
| --- | --- | --- | --- | --- |
| **2005** | January 14 | April 13 | 62 | Sailing only |
| **2006** | January 17 | February 11 | 21 | Whalewatch only |
| **2007** | January 28 | February 28 | 30 | Skiff and Whalewatch |
| **2008** | February 8 | May 8 | 75 | All |
| **2009** | January 11 | March 29 | 64 | Skiff and Whalewatch |
| **2010** | January 20 | April 18 | 72 | Sailing only |
| **2011** | March 3 | April 12 | 35 | RHIB only |
| **2012** | May 5 | June 6 | 31 | Sailing only |
| **2013** |  |  | 0 | NO EFFORT |
| **2014** | April 2 | May 12 | 26 | RHIB only |
| **2015** | February 11 | April 12 | 56 | RHIB and Sailing |
