# Supplementary material for: Critical Decline of the Eastern Caribbean Sperm Whale Population
Source: PLoS One. 2016 Oct 5;11(10):e0162019. doi: 10.1371/journal.pone.0162019 (PMC5051958; doi:10.1371/journal.pone.0162019)
Supplement: S2 Table — Details of field projects contributing opportunistic data. (DOCX) [file pone.0162019.s004.docx]

**Supplementary data:**

Supplementary photo-identification data were collected off several islands in the Lesser Antilles by eight different organizations across 33 years. Field methods were of three types based on platform (Table S2): research vessels dedicated to comprehensive sperm whale research, including photo-identification (D in Table S2), research vessel dedicated to cetacean abundance and distribution surveys, including photo-identification (S in Table S2); and opportunistic photo-identifications collected from whale watch vessels (W in Table S2).

The following organizations each contributed <5% of the total photo-identifications used in this analysis (in order of greatest contributions): Association Evasions Tropical (AET), Guadeloupe; Anchorage Whalewatch (AWW), Dominica; The International Fund For Animal Welfare (IFAW), USA; The Society for Dolphin Conservation (GRD), Germany; Woods Hole Oceanographic Institution (WHOI), USA; Ocean Research and Education Society (ORES), USA; and SeaWatch Foundation (SWF), UK. We thank the crew of RV ‘Rambler’; the crews of RV ‘Song of the Whale’; the crews of AET’s SV ‘Tzigane IV’; all the crews of the whale watch vessels off Dominica, in particular P. Charles and P. Francis, who collected the identifications used here; D. Perryman and the staff of Dive Dominica greatly helped SWF while in Dominica, as well as the IFAW crews during research. WHOI research was conducted under National Marine Fisheries Service Permit 573 and funding came from the Laurel Foundation, National Geographic Society, and National Science Foundation BNS 85-08047.

Results from work by Woods Hole Oceanographic Institution [1–5] and the International Fund For Animal Welfare [6] have been published. Data collected by researchers from Dalhousie University while in Guadeloupe in 2004 were collected in collaboration with Association Evasion Tropicale. No data were available from 1985 to 1989, 1992 to 1994, and 1997 to 1998.

Table S2 - Details of field projects contributing opportunistic data

| **Dates** | **Nearest**  **Island** | **Project Leader** | **Research**  **Group^a^** | **Type** | **# of**  **Photos** | **Individuals**  **Identified** |
| --- | --- | --- | --- | --- | --- | --- |
| 1981-1991 | Dominica | William Watkins | WHOI | D | 44 | 2 |
| 1984 | Dominica | George Nichols | ORES | D | 18 | 13 |
| 1995 | Dominica | Jonathan Gordon | IFAW | D | 218 | 59 |
| 1995 | Grenada | Jonathan Gordon | IFAW | D | 7 | 7 |
| 1996 | Dominica | Jonathan Gordon | IFAW | D | 81 | 36 |
| 2000 | Guadeloupe | Carole Carlson | IFAW | D | 7 | 6 |
| 1999 | Dominica | Peter Evans | SWF | W | 7 | 6 |
| 2000 | Guadeloupe | Caroline Rinaldi | AET | D/W | 9 | 8 |
| 2001 | Guadeloupe | Caroline Rinaldi | AET | D/W | 17 | 15 |
| 2002 | Guadeloupe | Caroline Rinaldi | AET | D/W | 11 | 9 |
| 2003 | Guadeloupe | Caroline Rinaldi | AET | D/W | 23 | 22 |
| 2005 | Guadeloupe | Caroline Rinaldi | AET | D/W | 275 | 75 |
| 2006 | Guadeloupe | Caroline Rinaldi | AET | D/W | 217 | 61 |
| 2007 | Guadeloupe | Caroline Rinaldi | AET | D/W | 170 | 46 |
| 2008 | Guadeloupe | Caroline Rinaldi | AET | D/W | 165 | 43 |
| 2009 | Guadeloupe | Caroline Rinaldi | AET | D/W | 6 | 4 |
| 2006 | Dominica | Petra Charles | AWW | W | 20 | 11 |
| 2007 | Dominica | Pernell Francis | AWW | W | 141 | 49 |
| 2008 | Dominica | Pernell Francis | AWW | W | 154 | 48 |
| 2009 | Dominica | Pernell Francis | AWW | W | 15 | 12 |
| 1999 | Dominica | Andrea Steffen | GRD | W | 7 | 3 |
| 2000 | Dominica | Andrea Steffen | GRD | W | 9 | 5 |
| 2001 | Dominica | Andrea Steffen | GRD | W | 48 | 14 |
| 2003 | Dominica | Andrea Steffen | GRD | W | 25 | 9 |
| 2004 | Dominica | Andrea Steffen | GRD | W | 11 | 6 |
| 2005 | Dominica | Andrea Steffen | GRD | W | 19 | 6 |
| 2006 | Dominica | Andrea Steffen | GRD | W | 18 | 6 |
| 2013 | St. Martin | Nicolas Maslach | AGOA | S | 4 | 1 |
| 2004 | Guadeloupe | Shane Gero | Dalhousie | W | 23 | 22 |

^a^ WHOI, Woods Hole Oceanographic Institution; ORES, Oceanic Research and Education Society; IFAW, International Fund for Animal Welfare; SWF, Sea Watch Foundation; AET, Association Evasion Tropicale; AWW, Anchorage Whale Watch; GRD, German Society for Dolphin Conservation; AGOA, Agoa Marine Mammal Sanctuary, French West Indies; Dalhousie, Dalhousie University. Type defines the research platform as either D, dedicated research vessel, S dedicated survey vessel, or W, whale watch.

^b^ Individuals identified are unique within each field season (row) but may be recounted between seasons or organizations.
