# Supplementary material for: Critical Decline of the Eastern Caribbean Sperm Whale Population
Source: PLoS One. 2016 Oct 5;11(10):e0162019. doi: 10.1371/journal.pone.0162019 (PMC5051958; doi:10.1371/journal.pone.0162019)
Supplement: S3 Table — Trends in numbers of adults, calves, adults+calves and calves/adults for each unit for which we had reliable estimates in 4 or more years (from linear regression) and overall (from linear mixed effects model), together with results of two-sided tests of null hypotheses that the overall trend is zero, and that units were equally likely to increase as decrease in size during the study period (sign test). The final three lines give the mean value of each measure over all years and units with available data, the overall percent trend and 95% confidence intervals. (DOCX) [file pone.0162019.s005.docx]

**Alternative Methods and Impacts on Results:**

Minimal number of years with estimates:

In the main text, we present the results which include all 16 units for which there were at least 2 reliable estimates of unit size in different years. The results below, in Table S3, demonstrate that the conclusions are very similar if only the 12 units for which 4 or more years with reliable estimates are included in the analysis.

Table S3 - Trends in numbers of adults, calves, adults+calves and calves/adults for each unit (from linear regression) and overall (from linear mixed effects model), together with results of two-sided tests of null hypotheses that the overall trend is zero, and that units were equally likely to increase as decrease in size during the study period (sign test). The final three lines give the mean value of each measure over all years and units with available data, the overall percent trend and 95% confidence intervals.

|  | Trend per year in | | | |
| --- | --- | --- | --- | --- |
| Unit | Adults | Calves | Adults+Calves | Calves/Adults |
| A | -0.315 | -0.165 | -0.480 | -0.015 |
| D | -0.528 | 0.000 | -0.528 | 0.037 |
| F | -0.420 | 0.122 | -0.299 | 0.041 |
| J | -0.146 | -0.003 | -0.149 | -0.001 |
| K | -0.275 | -0.242 | -0.516 | -0.040 |
| N | -0.262 | -0.214 | -0.476 | -0.036 |
| P | -0.300 | -0.600 | -0.900 | -0.055 |
| Q | -0.105 | -0.263 | -0.368 | -0.085 |
| R | -0.109 | 0.116 | 0.008 | 0.016 |
| S | 0.049 | -0.170 | -0.121 | -0.041 |
| T | 0.100 | 0.500 | 0.600 | 0.062 |
| U | -0.059 | -0.151 | -0.211 | -0.038 |
| All: | -0.195 | -0.071 | -0.264 | -0.010 |
| 95% CI | (-0.312 -0.078) | (-0.147 0.005) | (-0.423 -0.105) | (-0.022 0.002) |
| p (Trend) | 0.001 | 0.067 | 0.001 | 0.112 |
| p (Sign test) | 0.001 | 0.079 | 0.001 | 0.139 |
| Mean | 4.30 | 1.00 | 5.29 | 0.16 |
| Trend (%) | -4.55 | -7.11 | -4.99 | -6.20 |
| 95% CI | (-7.27 -1.82) | (-14.71 0.50) | (-7.99 -1.98) | (-13.87 1.48) |
