# Supplementary material for: Critical Decline of the Eastern Caribbean Sperm Whale Population
Source: PLoS One. 2016 Oct 5;11(10):e0162019. doi: 10.1371/journal.pone.0162019 (PMC5051958; doi:10.1371/journal.pone.0162019)
Supplement: S4 Table — Mark-recapture models fit to identifications of adult sperm whales in the eastern Caribbean, 1984–2015 sorted by decreasing ΔAIC. (DOCX) [file pone.0162019.s006.docx]

## **Overall mark-recapture population trend**

Here, we have updated the mark-recapture analysis described by Whitehead and Gero [7] to include data from 2014 and 2015. This analysis includes mortality, the possibility of heterogeneity in identifiability, and also for different types of trend in population size with time. We fitted models using by maximum likelihood methods and confidence intervals from the likelihood support function. The fit of different models to the data is compared using the Akaike Information Criterion (AIC).

The population models strongly supported the inclusion of heterogeneity in identification (Table S4). The best fitting population model included an increase in population at 5.1%/yr (95% CI: 2.9%/yr to 7.3%/yr) until reaching 279 (95% CI: 238-336) individuals in 2009 (95% CI: 2007-2013), and then remaining stable until 2015 (top row of Table S4). Also well supported (ΔAIC = 1.13; second row Table S5), is a model with an increasing population (5.3%/yr.; 95% CI: 2.9%/yr to 7.5%/yr) until 2009 (95% CI: 2007-2014) and then a decrease until 2015 (-2.7%/yr.; 95% CI: -32.2%/yr. to +2.9%/yr).

**Table S4 - Mark-recapture models fit to identifications of adult sperm whales in eastern Caribbean, 1984-2015 sorted by descending ΔAIC.**

| **Heterogeneity in identifications?** | **Model** | **Population trend** | **AIC** | **ΔAIC** |
| --- | --- | --- | --- | --- |
| Yes | Increase then level |  | 2205.96 | 0.00 |
| Yes | Broken stick |  | 2207.09 | 1.13 |
| Yes | Trend (quadratic) |  | 2208.12 | 2.16 |
| Yes | Trend |  | 2209.43 | 3.47 |
| Yes | Two steps |  | 2212.28 | 6.32 |
| Yes | Step |  | 2212.81 | 6.85 |
| Yes | Stable |  | 2220.99 | 15.03 |
| No | Trend |  | 2373.67 | 167.71 |
| No | Stable |  | 2379.26 | 173.3 |
