# Supplementary material for: Critical Decline of the Eastern Caribbean Sperm Whale Population
Source: PLoS One. 2016 Oct 5;11(10):e0162019. doi: 10.1371/journal.pone.0162019 (PMC5051958; doi:10.1371/journal.pone.0162019)
Supplement: S5 Table — Models of Survival in best-studied social units, 2005–2015. (DOCX) [file pone.0162019.s007.docx]

**Survival of well-studied units using maximum likelihood:**

We also estimated survival within these well-studied units using data for just those years when we are reasonably confident that all members were identified (the same criteria as outlined above) using maximum likelihood. If, for a particular social unit observed in years *t*_1_ and *t*_2_ but with no observations in intermediate years, *n*_1,2_ animals were identified in both years and *n*_1,-2_ in the first but not the second, then the log-likelihood of these data are (within a constant):

$$L=n_{1,2}\sum_{t=t_{1}+1}^{t_{2}} Log\left( s\left( t \right) \right){+n}_{1,-2}Log\left[ \sum_{t=t_{1}+1}^{t_{2}} (1-s\left( t \right))\prod_{x=t_{1}+1}^{t-1} s\left( t \right) \right]$$

where *s*(*t*) is the survival rate between year *t*-1 and year *t*. Then the log-likelihood of the data is obtained by summing *L* over all units and successive pairs of yearswhen each unit was well identified, and estimates of *s* are obtained by maximizing this likelihood. We compared the utility of a variety of functions for *s* using AIC. These estimates of *s* will generally underestimate true survival as they include dispersal of maturing males, as well as the rare occasions when animals switch units.

While the best model of survival included a separate estimate for each year (S5 Table, next page), these separate annual estimates were too imprecise to be much useful. Thus, in Fig 2 of the main text we present biannual estimates of survival. Confidence limits are from 1000 bootstrap replicates of unit identification histories.

S5 Table - Models of survival in best-studied social units, 2005-2015.

| Model | Survival trend | AIC | ΔAIC |
| --- | --- | --- | --- |
| Every year | ^. .^ . ^.^ _. ._ . . ^.^ . | 294.23 | 0.00 |
| Each two years* |  | 305.71 | 11.48 |
| Stable |  | 312.49 | 18.26 |
| Two steps |  | 312.89 | 18.66 |
| Trend (quadratic) |  | 309.83 | 15.60 |
| Trend |  | 314.43 | 20.20 |
| Step |  | 316.41 | 22.18 |

* Presented in Fig 2 of the main text
